# The influence of reward and loss outcomes after free- and forced-tasks on voluntary task choice

**DOI:** 10.1007/s00426-024-02009-9

**Published:** 2024-07-30

**Authors:** Victor Mittelstädt, Ian G. Mackenzie, Hartmut Leuthold

**Affiliations:** University of Tübingen, Tübingen, Germany

## Abstract

In four experiments, we investigated the impact of outcomes and processing mode (free versus forced) on subsequent voluntary task-switching behavior. Participants freely chose between two tasks or were forced to perform one, and the feedback they received randomly varied after correct performance (reward or no-reward; loss or no-loss). In general, we reasoned that the most recently applied task goal is usually the most valued one, leading people to prefer task repetitions over switches. However, the task values might be additionally biased by previous outcomes and the previous processing mode. Indeed, negatively reinforcing tasks with no-reward or losses generally resulted in more subsequent switches. Additionally, participants demonstrated a stronger attachment to free- compared to forced-tasks, as indicated by more switches when the previous task was forced, suggesting that people generally value free over forced-choice task goals. Moreover, the reward manipulation had a greater influence on switching behavior following free- compared to forced-tasks in Exp. 1 and Exp. 3, suggesting a stronger emphasis on evaluating rewarding outcomes associated with free-task choices. However, this inflationary effect on task choice seemed to be limited to reward and situations where task choice and performance more strongly overlap. Specifically, there was no evidence that switching behavior was differentially influenced after free-and forced-task as a function of losses (Exp. 2) or reward when task choice and task performance were separated (Exp. 4). Overall, the results provide new insights into how the valuation of task goals based on choice freedom and outcome feedback can influence voluntary task choices.

## Introduction

The capacity to regulate behavior in environments characterized by multiple sources of information is crucial for adaptive functioning. On the one hand, effective behavior necessitates the ability to maintain stable information processing aligned with the current task goal. On the other hand, it is equally vital to continuously monitor both one's own behavior and the environment for cues that signal the potential benefits of switching to an alternative task goal. Unraveling the cognitive mechanisms that enable us to navigate these contrasting demands effectively represents a significant challenge in cognitive psychology (e.g., Dreisbach & Fröber, 2019; Eppinger et al., 2021; Goschke, 2000).

In most cases, the achievement of a task goal entails engaging in demanding cognitive processes, such as studying to pass an exam. In the field of cognitive psychology, the concept of goals as mental representations that guide our behavior has typically been investigated in laboratory settings through the notion of task-sets, which encompass the rules that participants need to follow in order to successfully link stimuli to responses (e.g., Kiesel et al., 2010; Koch et al., 2018; Meiran, 2010; Monsell, 2003; Schumacher & Hazeltine, 2016). For example, participants may be instructed to perform a letter task, wherein they determine whether a letter is a vowel or consonant and respond accordingly. Even the execution of such seemingly straightforward tasks requires cognitive effort, and research indicates that individuals weigh the costs and benefits associated with expending cognitive resources when deciding how to perform a task (e.g., how much effort to invest in studying for the exam or completing the letter task) (e.g., Shenhav et al., 2013, 2021). When deciding *which* of multiple tasks to choose, individuals often exhibit a bias towards perseveration, wherein they favor repeating the same task (e.g., Arrington & Logan, 2004; Koch et al., 2018; Kool et al., 2010). Based on the notion that people choose the task with the highest value, this indicates that switch task goals are less valued than the repetition task goals, because of the temporal costs (e.g., Mittelstädt et al., 2019, 2021) and the involved cognitive effort (e.g., Kool et al., 2010; Mendl & Dreisbach, 2022) when deciding to switch tasks.

In the present study, we are investigating whether and how outcomes (rewards and losses) after choosing and performing a task can further modulate the value of task goals to bias subsequent task choice behavior. Many studies have demonstrated that task performance-related control signals can be modulated by receiving performance-contingent or -independent rewards to bias subsequent task processing (e.g., Braem et al., 2012; Stürmer et al., 2011; van Steenbergen et al., 2009; Yamaguchi & Nishimura, 2019), presumably because receiving rewards can strengthen recently activated task goals (e.g., Umemoto & Holroyd, 2015), aligning with Thorndike's (1927) law of effect. Moreover, several studies have shown that when people choose between different options (e.g., different cards in gambling-like situations), they are more likely to repeat the same option following a reward and switch to another option following a loss (e.g., Elston et al., 2021; Worthy et al., 2013). Thus, although we are not aware of any studies investigating the effects of recent outcomes when people decide between different cognitive tasks rather than different options in gambling-like situations, it is probably not surprising if receiving a reward (compared to no reward) also provides choice-related signals that bias subsequent task choices. Specifically, we hypothesize that when people receive rewards for correct performance, they will be less likely to switch away from the current task in the subsequent trial compared to a condition without rewards. Conversely, receipt of losses (compared to no losses) will increase the likelihood of switching to the other task.

While previous studies have investigated the influence of prior reward cues and learning about specific behavior-reward associations on voluntary task choice behavior (e.g., Braun & Arrington, 2018; Fröber & Dreisbach, 2016; Spitzer et al., 2024), to our knowledge, no study to date has examined the immediate effects of outcome reception on subsequent voluntary task choices without any explicit reward cues or prior learning phases. For example, Fröber and Dreisbach (2016) have examined the motivating effects of reward anticipation on voluntary behavior by providing cues signaling the possibility of low or high (task-unspecific) rewards before each trial. Moreover, Braem (2017) demonstrated that participants who received greater rewards for task switches compared to task repetitions during a learning phase exhibited higher levels of switching behavior in a subsequent test phase, suggesting reinforcement learning of more abstract (task-unspecific) components related to cognitive flexibility.

**Fig. 1:**
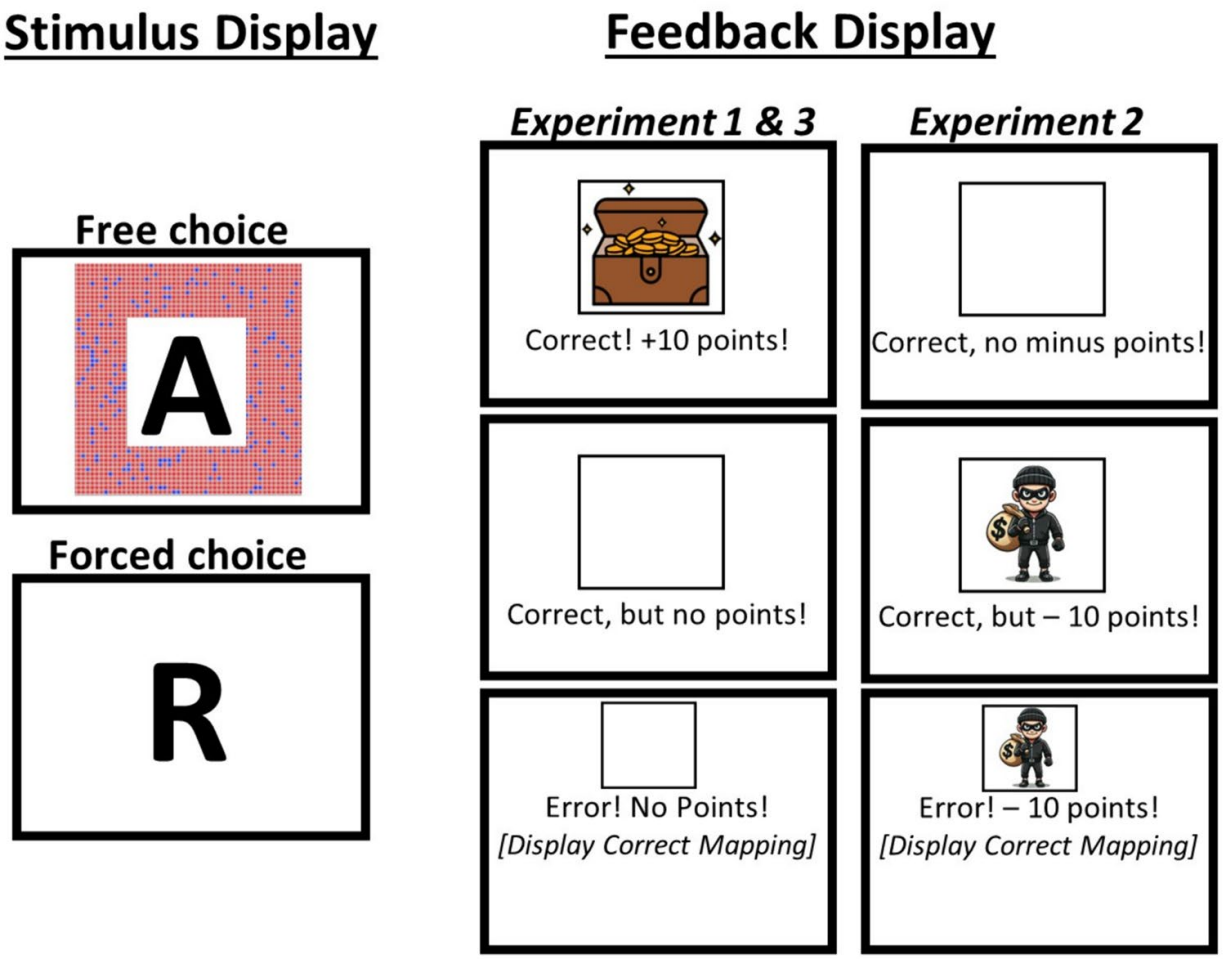
Sketch of the stimulus display and the experiment-specific feedback display (not to scale) in Experiment 1 and 3 (reward versus no-reward) and Experiment 2 (loss versus no-loss). Note that Experiment 3 was similar to Experiment 1, but the intertrial interval duration after feedback presentation was additionally varied. The image of the thief was created using DALL-E by OpenAI. Note that this image of the thief was not the one used in the experiment (see text for more details)

Importantly, the present study goes beyond these prior studies by considering that people may differentially value free-choice compared to forced-choice task goals. In general, the investigation of voluntary task choice behavior can be approached using various experimental methods (e.g., Arrington & Logan, 2004, 2005; Brosowsky & Egner, 2021; Brüning et al., 2020; Dreisbach & Jurczyk, 2022; Jurczyk et al., 2019, 2021; Mittelstädt et al., 2022, 2023; Mittelstädt et al., 2018a, b; Spitzer et al., 2022; Wong et al., 2022). Critically, in the present study, we employed the hybrid free-forced choice paradigm introduced by Fröber and Dreisbach (2017). This paradigm involves the intermixing of free and forced choice trials, where participants are presented with either one task stimulus (forced choice) or two task stimuli (free choice) (see Fig. 1). In a modified version of this paradigm, Qiao et al. (2022) reported additional performance costs between free and forced-choice trials, even when repeating the same task, suggesting that free and forced-task goals may be differentially internally represented. Moreover, they reported a larger task repetition bias following free-choice trials compared to forced-choice trials, suggesting that participants are less inclined to disengage from a task goal that was freely chosen. This could indicate that choosing a task is itself rewarding and, hence, that a differential valuation of freely versus forced-chosen task goals can bias subsequent choice behavior. While Mittelstädt et al. (2024) have recently shown that the findings by Qiao et al. (2022) cannot be explained by the specific cues used, the reciprocal relationship between processing modes and behavioral outcomes was not investigated.^1^

Specifically, while we generally predict that people are more likely to switch away from a task when experiencing negative outcomes (no reward or loss) than positive outcomes (reward or no loss), and when they have not chosen the task themselves, it is also possible that these two choice biases interact rather than being additive. Drawing on theoretical proposals and studies from various research fields (e.g., Cockburn et al., 2014; Mühlberger et al., 2017; Leotti et al., 2010; Yeung et al., 2005; Zhou et al., 2010), it seems possible that individuals pay more attention to the outcomes of tasks they have freely chosen compared to those that were imposed on them. Moreover, and not mutually exclusive, people may inflate the value of outcomes associated with freely-chosen task goals as they might believe their personal choice led to the improved outcome, even though positive and negative outcomes were randomly delivered for correct performance. For example, Yeung et al. (2005) observed increased neural outcome monitoring in a simple gambling task (i.e., selecting between different colored circles) when participants made active choices rather than when a choice was made for them. Although we are not aware of any studies investigating the interplay between task processing mode (free vs. forced) and outcomes (e.g., reward vs. no reward) on subsequent voluntary task choice behavior, these previous studies suggest that outcome manipulations might have a greater influence on switching behavior following free- compared to forced-task conditions.

## Experiment 1

In this experiment, participants freely chose between two tasks (letter and color) in half of the trials (i.e., two stimuli were presented), whereas in the other half of trials, they were forced to perform a task (i.e., only one stimulus was presented). As is common in this and other voluntary task-switching paradigms, each task was assigned to a specific hand, allowing task choice to be identified based on the response made (e.g., Arrington & Logan, 2004; Fröber & Dreisbach, 2016; Liu & Yeung, 2020; Mittelstädt et al., 2022). Critically, participants randomly received reward or no reward after correctly performing a task. Our focus was to examine which task participants voluntarily selected in free choice trials (i.e., the task associated with repetitions or switches) as a function of previous task mode (free versus forced) and previous trial feedback (reward versus no-reward). We hypothesized that participants will be more likely to switch after a forced-chosen as opposed to a freely-chosen goal and that they will be more likely to switch after having received no-reward compared to reward (i.e., main effects of previous mode and feedback). Furthermore, we tested whether participants’ sensitivity to obtaining reward (versus no reward) is larger after a freely-chosen as compared to a forced-chosen task.

### Method

#### Participants

As preregistered,^2^ 50 participants were tested online. In this and the next experiment, all participants provided informed consent and could receive course credits for their participation. Furthermore, all experiments adhered to the standards set by the local ethics committee and were performed in accordance with the ethical standards described in the 1964 Declaration of Helsinki. Following our preregistration, data from seven participants were excluded due to excessively high repetition rates in correct trials after our data preparation procedure (> 95% task repetitions). Note that in this and the following experiments, the result pattern were similar when including all participants or when applying stricter criteria for excluding participants with extreme task choice behavior (i.e., > 90% task repetition and > 90% task switches). The remaining 43 participants (36 female, 40 right-handed) ranged in age from 18 to 26 years (M = 20.72 years).

#### Apparatus and stimuli

The experiment was conducted online using the JavaScript library jsPsych (De Leeuw, 2015). All visual stimuli were presented on a grey background, with a centrally positioned black plus sign serving as the fixation point. For the letter task, participants had to classify whether a letter was a vowel or consonant. The possible stimuli included the letters A, E, I, U, G, K, M, or R. For the color task, participants had to classify whether a set of dots was primarily red or blue (see Fig. 1). The proportion of red and blue dots was always the same (80% versus 20% or vice versa), but the specific arrangement of the dots was randomized for each trial. The tasks were assigned to the index and middle fingers of either the left hand (mapped to the “Q” and “W” keys) or the right hand (mapped to the “O” and “P” keys). The task-to-hand mapping was counterbalanced across participants, and the specific stimulus–response mapping was randomly selected for each participant.^3^ The presence of a colored treasure box indicated whether a reward was obtained.

#### Procedure

Participants were tested in eight blocks, with 100 trials per block, resulting in a total of 800 trials. Each experimental block consisted of a random mixture of 50% free choice trials and 50% forced choice trials (i.e., 25% forced-color and 25% forced-letter trials). The specific stimuli for each task were randomly selected for each trial (i.e., stimulus repetitions were possible).

At the beginning of each trial, a fixation cross appeared on the screen for 500 ms. Subsequently, either two stimuli (for free choice trials) or one stimulus (for forced choice trials) was presented and remained on the screen until the participant responded. After a correct response, it was randomly determined whether participants received a reward (indicated by the presentation of a treasure box and the message “Correct! + 10 points!”) or no reward (no treasure box and the message “Correct, but no points!”). Thus, overall, participants were rewarded in approximately 50% of correct trials (i.e., *approximately* 50% because the probability of receiving a reward after each correct trial was consistently 50%, irrespective of the previous proportion of correct trials). In the case of incorrect responses, participants received the message “Error! No points!” displayed centrally. The feedback screen was always presented for 1000 ms after a response was registered, followed by a blank interval of 500 ms before the next trial began.

Participants received written instructions only. Specifically, they were instructed that in free choice trials (when both stimuli required a response), they were free to choose whichever task they wanted to perform. However, in forced choice trials (when only one stimulus appeared), they were instructed to perform the task corresponding to the stimulus. Additionally, they were informed that they would occasionally receive points after correct responses, but no further information about the reward structure was provided. To give the points some significance, participants were informed that the top 20% of participants with the highest points would receive an additional 10€ voucher. Furthermore, participants were falsely informed that the experiment would consist of a maximum of 11 blocks, but after the eighth block, the experiment would be shortened based on the number of points collected up to that point. However, in reality, all participants were informed after the eighth block that they had collected enough points for the experiment to conclude. We reasoned that this instruction, along with the chance to win a voucher, would further motivate participants to pay attention to the outcomes/points obtained. Note that vouchers and the untruthful instruction regarding a maximum of 11 blocks were used in all experiments.

#### Data preparation

The practice blocks and the first trial of each block were excluded from any analyses. The task performed on each trial was categorized based on the response hand. Subsequently, trials were categorized as repetition or switch trials based on the task performed on trials n and n – 1. We then excluded trials with RTs less than 200 ms (0.04%) or more than 3000 ms (0.4%), error trials (4.8%), and trials following incorrect responses (4.9%) for the analyses on switch percentages reported in the main text. Note that the task choice results in all experiments were very similar when error trials were included. The same outlier criteria were used for the exploratory analyses on task performance reported in Appendix A.

### Results and discussion

**Fig. 2:**
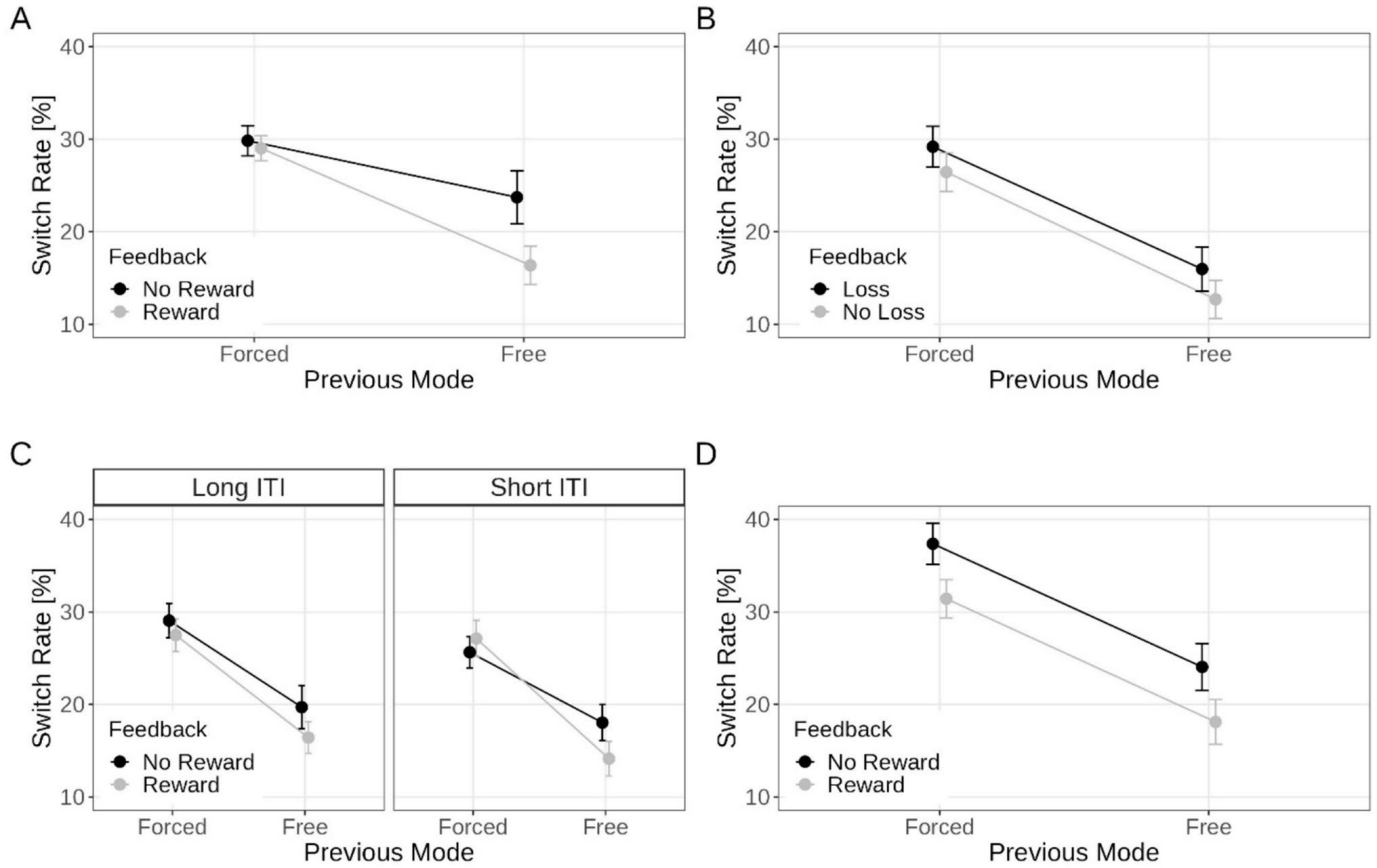
Percentage of task switches as a function of previous mode (free, forced) and feedback (Experiment 1 and 3 and 4: reward, no-reward; Experiment 2: loss, no-loss) separately for Experiment 1 (panel **A**), Experiment 2 (panel **B**), Experiment 3 (panel **C**) and Experiment 4 (panel **D**). Because, in Experiment 3, the intertrial interval (ITI) was additionally varied, Panel **C** plots the corresponding percentage of switch rates as a function of short versus long durations

Figure 2A illustrates the percentage of task switches based on the previous mode (free versus forced) and feedback (reward versus no reward). A 2 × 2 within-subject ANOVA revealed a significant main effect of previous mode, indicating a higher likelihood of switching away from a forced-chosen task compared to a freely-chosen task (29.4% and 20.0%), *F*(1, 42) = 9.68, *p* = 0.003, η_p_^2^ = 0.19. The main effect of feedback was also significant, with increased switching observed after the absence of reward compared to receiving a reward (26.8% and 22.7%), *F*(1, 42) = 15.45, *p* < 0.001, η_p_^2^ = 0.27. Interestingly, a significant interaction was observed, *F*(1, 42) = 12.27, *p* = 0.001, η_p_^2^ = 0.23. As depicted in Fig. 2A, participants exhibited a strong bias towards increased switches after receiving no reward compared to receiving a reward for a freely-chosen task (23.7% vs 16.4%; with *p* < 0.001 for the pairwise comparison), while no significant reward-related difference was observed after a forced-chosen task (29.8% vs 29.0%, with *p* = 0.506).

## Experiment 2

Experiment 1 showed on average fewer task switches following a reward compared to when no reward was received. Additionally, participants showed a general reluctance to switch away from a freely chosen task goal, indicating a preference for tasks that were freely selected. Furthermore, the influence of the reward manipulation was more pronounced (and only significant) following freely-chosen tasks compared to forced-chosen tasks, suggesting that participants placed greater emphasis on receiving rewards for tasks they had freely chosen. The goal of Experiment 2 was to determine whether these findings would replicate when using a manipulation involving losses (versus no losses) as outcomes.

### Method

#### Participants

As preregistered, a new sample of 50 participants was tested in this experiment. Following the same data preparation procedure, data from eight participants were excluded due to having more than 95% task repetitions. Additionally, data from two additional participants were excluded due to their clear inability to properly perform the experiments (accuracy below 60%). The final analysis included data from 40 participants (32 female, 34 right-handed) ranging in age from 19 to 46 years, with a mean age of 22.3 years.

#### Apparatus, stimuli and procedure

The methodological aspects were the same as in Experiment 1, with the exception that in this experiment, participants now randomly lost −10 points after correct trials instead of receiving no points (see Fig. 1). Additionally, participants always lost −10 points after erroneous responses. To highlight the losses, a picture of a thief was presented. The picture used was retrieved from www.pixabay.com (https://pixabay.com/illustrations/thief-steal-thieve-criminal-crook-3306100/). To illustrate the image of a thief for Fig. 1, a picture was created using DALL-E by OpenAI. Each participant started with 1000 points and was informed that the top 20% with the fewest losses would receive a 10€ voucher.

#### Data preparation

We followed the same data preparation procedure. Hence, we excluded the first block and the first trial of each block. Furthermore, we excluded trials with RTs less than 200 ms (0.01%) or more than 3000 ms (0.4%), error trials (4.1%) and trials following incorrect responses (4.1%).

### Results and discussion

Figure 2B illustrates the percentage of task switches based on the previous mode (free versus forced) and feedback (loss vs no loss). Consistent with Experiment 1, the ANOVA revealed a significant main effect of previous mode, indicating a higher likelihood of switching away from a forced-chosen task compared to a freely-chosen task (27.8% and 14.3%), *F*(1, 39) = 31.08, *p* < 0.001, η_p_^2^ = 0.44. Furthermore, a significant main effect of feedback was again observed, with increased switching observed after experiencing a loss compared to no loss (22.6% and 19.6%), *F*(1, 39) = 11.08, *p* = 0.002, η_p_^2^ = 0.22. However, contrary to Experiment 1,^4^ there was no significant interaction, *F*(1, 39) = 0.12, *p* = 0.727, η_p_^2^ < 0.01. As depicted in Fig. 2B, participants exhibited similar influences of feedback obtained for both forced-chosen tasks (with *p* = 0.002) and freely-chosen tasks (with *p* = 0.028).

## Experiment 3

Experiment 2 revealed that not only reward versus no-reward, but also loss versus no-loss outcomes can influence subsequent task choice behavior by showing more task switches after loss compared to no loss. Furthermore, participants showed again a general preference for freely-chosen task goals over forced-chosen task goals, as they were more likely to switch away from a forced-chosen task goal. Contrary to Exp. 1, there was no evidence that the influence of the loss manipulation differentially impacted freely-chosen and forced-chosen tasks. While this suggests that rewards and losses, at least partially, differentially influence task choice behavior, one may also argue that the interaction in Exp. 1 reflects a false positive (Type 1 error). As this interaction seems theoretically interesting, we decided to conduct another experiment with a reward manipulation to see whether we would replicate the pattern observed in Experiment 1. Thus, Experiment 3 was similar to Experiment 1, except that we additionally varied the time after feedback presentation (intertrial interval short vs long).

The intertrial interval (ITI) is often varied in task-switching studies, and previous research (without outcome manipulations) has demonstrated increased switching behavior after a long compared to a short SOA (e.g., Arrington & Logan, 2005; Mittelstädt et al., 2019). Thus, observing a similar effect in the present study would more strongly connect the current task environment to the previous ones. Moreover, it also allows one to gain insights into the temporal dynamics of previous trial characteristics influencing subsequent choice behavior. Specifically, we wanted to explore whether the impact of the previous mode and previous feedback decays over time or remains relatively stable. Previous task-switching studies have suggested that the activation of previously applied task goals decays over time (e.g., Altmann & Gray, 2002). Additionally, several decision-making studies using neural networks have demonstrated that the influence of previous decisions decreases over time due to a decay of neural activation (e.g., Bonaiuto et al., 2016; Rustichini & Padoa-Schioppa, 2015; Urai et al., 2019). Since both positive outcomes and freely chosen tasks may enhance task goal values, their effects may also decay over time. However, participants may employ additional control processes to counteract this decay, enabling them to use this information in subsequent trials when facing another potentially effortful decision between tasks.

### Method

#### Participants

As preregistered, a new sample of 60 participants was tested in this experiment. Following the same data preparation procedure, data from 8 participants were excluded due to having more than 95% task repetitions. The final analysis included data from 52 participants (36 female, 45 right-handed) ranging in age from 18 to 30 years, with a mean age of 21.6 years.

#### Apparatus, stimuli and procedure

The methodological aspects were the same as in Experiment 1, with the exception that in this experiment, we additionally varied the intertrial interval (ITI) after feedback was presented (short ITI: 300 ms; long ITI: 1100 ms).

#### Data preparation

We again excluded the first block and the first trials of each block. We then excluded again trials with RTs less than 200 ms (0.04%) or more than 3000 ms (0.6%), error trials (4.2%) and trials following incorrect responses (4.2%).

### Results and discussion

Figure 2C illustrates the percentage of task switches based on the previous mode (free versus forced), feedback (reward versus no reward) and ITI (short versus long). Consistent with the previous two experiments, the ANOVA revealed a significant main effect of previous mode, indicating a higher likelihood of switching away from a forced-chosen task compared to a freely-chosen task (27.3% and 21.3%), *F*(1, 51) = 18.92, *p* < 0.001, η_p_^2^ = 0.27. Furthermore, a significant main effect of ITI was observed, with increased switching when the ITI was long compared to short (23.17% and 21.24%), *F*(1, 51) = 7.93, *p* = 0.007, η_p_^2^ = 0.13. The main effect of feedback was marginally significant, with increased switching observed after the absence of reward compared to receiving a reward (23.1% and 21.3%), *F*(1, 51) = 3.39, *p* = 0.071, η_p_^2^ = 0.06. However, the impact of feedback was significantly modulated by previous mode, *F*(1, 51) = 5.32, *p* = 0.025, η_p_^2^ = 0.09. As in Experiment 1, participants exhibited a strong bias towards increased switches after receiving no reward compared to receiving a reward for a freely-chosen task (18.9% vs 15.3%, with *p* = 0.014 for the pairwise comparison), while no reward-related significant difference was observed after a forced-chosen task (27.3% vs 27.3%, with p = 0.926). The two-way interactions involving ITI (both *p* > 0.291, η_p_^2^ < 0.03) and the three-way interaction (*p* = 0.108, η_p_^2^ < 0.05) were not significant.

## Experiment 4

**Fig. 3:**
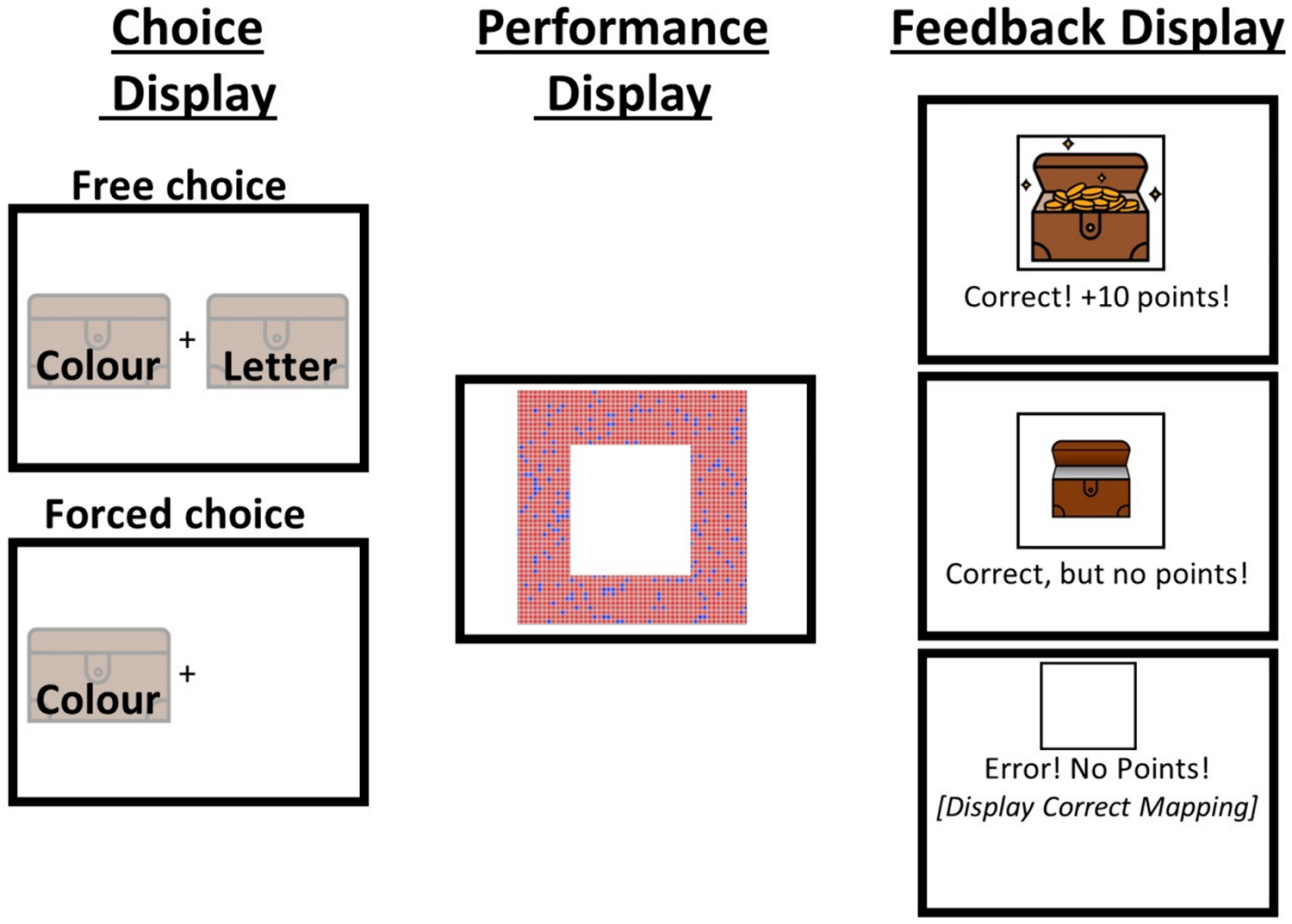
Sketch of the task choice and task performance displays and the feedback display (not to scale) in 4. See text for more details

Experiment 3 replicated the pattern observed in Experiment 1: Participants specifically preferred to repeat freely-chosen tasks compared to forced-chosen tasks and they specifically biased their task choice behavior based on reward versus no-reward outcomes for freely-chosen tasks. These influences on free-choice behavior were, if any, little influenced little by the time after feedback was presented. The goal of Experiment 4 was to determine whether participants would be similarly affected by the reward manipulation and previous mode when separating task choice from task processing. Specifically, in contrast to the previous experiments, participants in Experiment 4 could first choose which of two tasks they wanted to perform (two task cues) or were forced to choose (one task cue) before the corresponding task stimulus was presented (double-registration procedure, see Fig. 3). It is difficult to predict whether the previous pattern will replicate or not, as there are good arguments for both alternatives. For example, the reinforcing effects of freely-choosing tasks and obtaining rewards (as well as their interaction) could also be observed with this approach if these effects primarily arise due to changes in higher-level goal representations rather than lower-level perceptual and/or motor processes associated with task stimuli and responses. However, without seeing the actual task they had to perform with a double registration procedure and by temporally separating choice and task processing, participants might feel less attached to task goals and perceive they have less control over outcomes—a prerequisite that may be critical to observe biases in choice behavior.

As the belief in control over the (random) reward manipulation might influence choice behavior, we also explicitly asked participants to which extent they believed that the reward was contingent on their task choices and additionally measured via a questionnaire their individual locus of control, that is, how strongly they attribute outcomes in life to their own actions rather than external forces (e.g., Rotter, 1966; Stolz et al., 2020).

### Method

#### Participants

As preregistered, a new sample of 60 participants was tested in this experiment. Following the same data preparation procedure, data from six participants were excluded due to having more than 95% task repetitions. Moreover, one additional participant was excluded due to low accuracy (< 60%). The final analysis included data from 53 participants (44 female, 50 right-handed) ranging in age from 18 to 43 years, with a mean age of 21.6 years.

#### Apparatus, stimuli and procedure

The methodological aspects were the same as in Experiment 1, unless stated otherwise. In contrast to the previous experiments, the task choice and task performance screens were separated in this experiment (see Fig. 3). In total, there were 8 blocks with 96 trials each.

During the task choice phase, one (free-choice) or two (forced-choice) pictures of closed treasure boxes with the task cues “Letter” or “Color” were presented on the right and left sides of the screen. Participants could choose (or were forced to choose) one of the tasks with their middle and index fingers of their left hand by pressing the “S”- or “D”-key. The position of task-specific treasure boxes was fixed throughout the experiment but counterbalanced across participants. After a task was chosen, a black frame around the selected task treasure box was presented for 250 ms.

Following this, either a letter or color stimulus was presented, and participants performed the task by responding with the index and middle fingers of their right hand, pressing the “K”- or “L”-key, respectively. The task-specific response mapping was randomly selected for each participant. While we used the same tasks as in the previous experiment, only one stimulus was presented during the task performance screen in both free and forced choice trials.

After a correct response, it was randomly determined whether participants received a reward, indicated by the presentation of an open and filled treasure box and the message “Correct! + 10 points!”, or no reward, shown by an open but empty treasure box and the message “Correct, but no points!” In the case of incorrect responses, participants received the message “Error! No points!” displayed centrally. The feedback screen was always presented for 750 ms after a response was registered, followed by a blank interval of 500 ms before the next trial began.

After completing all the trials, participants were asked to fill out the IE-4 questionnaire (Kovaleva, 2012) in German, indicating their agreement with four items on a 5-point Likert scale. This questionnaire assessed the internal locus of control with two items, reflecting beliefs that life events can be controlled by active behavior: “If I work hard, I will succeed.” and “I'm my own boss.” The external locus of control was measured with the other two items, representing beliefs that life is controlled by external forces: “Fate often gets in the way of my plans.” and “Whether at work or in my private life, what I do is mainly determined by others.” Finally, participants were asked to rate how much they felt the reward was contingent on their choices during the experiment on a 4-point scale ranging from “Completely” (4) to “not at all” (1).

#### Data preparation

We again excluded the first block and the first trials of each block. We then excluded RTs related to the task performance screen (performance-RTs) with less than 200 ms (1.4%) or more than 3000 ms (0.4%), error trials (6.3%) and trials following incorrect responses (6.3%). Moreover, as we also measured reaction times related to the task choice screen (choice-RTs), we additionally excluded choice-RTs (0.7%) with exceptionally high durations (> 2000 ms, based on visual inspection).

### Results and discussion

Figure 2D illustrates the percentage of task switches based on the previous mode (free versus forced) and feedback (reward versus no reward). A 2 × 2 within-subject ANOVA revealed a significant main effect of previous mode, indicating a higher likelihood of switching away from a forced-chosen task compared to a freely-chosen task (34.4% and 21.1%), *F*(1, 52) = 53.37, *p* < 0.001, η_p_^2^ = 0.51. The main effect of feedback was also significant, with increased switching observed after the absence of reward compared to receiving a reward (30.7% and 24.8%), *F*(1, 52) = 9.59, *p* = 0.003, η_p_^2^ = 0.16. The interaction was not significant (*p* > 0.99, η_p_^2^ < 0.01). As depicted in Fig. 2D, participants switched more often after receiving no reward compared to receiving a reward for both freely-chosen task (with *p* = 0.015 for the pairwise comparison) and forced-chosen task (*p* = 0.001).

#### Exploratory analyses related to questionnaire data

The majority of the 53 participants indicated that the reward was rather not contingent on their choices during the experiment, as 15 participants responded with (1), and 31 participants responded with (2) on the 4-point scale ranging from “Completely” (4) to “not at all” (1). Six participants responded with (3), and one participant responded with (4). Reanalyzing the data, excluding the data of 15 participants who selected (1), revealed only significant main effects (with *p*s < 0.008), but no significant interaction (*p* = 0.809). When considering only the seven participants who responded with (3) or (4), there were descriptively more switches after no-reward compared to reward following free-task choices (a difference of 21%) than after forced-task choices (a difference of 17%), but meaningful statistical analyses are obviously not possible with this low sample size.

The internal locus mean score of the two items was generally high, as indicated by an average value of 4.1 on the 5-point Likert scale, with only 11 participants having values less than 4. Reanalyzing the data, excluding the data of the latter 11 participants, revealed only significant main effects (with *p*s < 0.005), but no significant interaction (*p* = 0.439). The external locus mean score of the two items was rather low, as indicated by an average value of 2.2 on the 5-point Likert scale, with only seven participants having values larger than 2.5. Reanalyzing the data, excluding the data of the latter seven participants, revealed only significant main effects (with *p*s < 0.011), but no significant interaction (*p* = 0.751).

In sum, while the exploratory questionnaire-based data analyses did not provide any evidence that control beliefs of outcomes (related to the specific manipulation or in general) modulated how participants adapted their choices in the present task environments, some caution needs to be applied with this interpretation, as these control beliefs were generally quite low for the majority of participants.

## Additional task choice analyses of Experiment 1–4

The results of all four experiments demonstrate that participants are less likely to switch away from a free-choice task compared to a forced-choice task (i.e., main effects of previous mode on switch rates). One possible explanation for this preference is that participants have an overall preference for one of the two tasks. Consequently, they might tend to select that particular task more frequently in free-choice trials. In situations where they encounter two consecutive free-choice trials, they may opt for the same task both times due to their overarching preference for it, thereby increasing repetition rates. On the contrary, when the preceding trial involved a forced-choice task, it could have been either the preferred or the less favored task. As a result, choosing the preferred task in the next trial would lead to smaller repetition rates. To investigate this possibility, we categorized each participant as either having an “overall color preference” or an “overall letter preference.” This categorization allowed us to classify the previous task response in each trial as either the “preferred task” or the “nonpreferred task.” We hypothesized that if participants are still more likely to switch away after a forced-choice trial than a free-choice trial when controlling for the fact that the previous task for both modes was either the preferred or nonpreferred one, an overall task preference account cannot explain the specific free-choice repetition bias.

Indeed, when the previous task was the nonpreferred one, participants were in all experiments more likely to switch away after a forced-choice trial than after a free-choice one (Exp. 1: 47.4% vs 39.6%, *p* = 0.092; Exp. 2: 44.8% vs 33.6%, *p* = 0.001; Exp. 3: 39.3% vs 28.5%, *p* = 0.002, Exp. 4: 48.5% vs 34.1%, *p* < 0.001). Moreover, in Exp. 2–Exp. 4, when the previous task was the preferred one, participants were at least descriptively more likely to switch away after a forced-choice trial than after a free-choice one (Exp. 2: 11.8% vs 9.7%, *p* = 0.084; Exp. 3: 15.6% vs 13.0%, *p* = 0.127; Exp. 4: 21.1% vs 17.5%, *p* = 0.008). Thus, while an overall stable preference for one task certainly contributes to the free-task repetition bias, it seems not fully explanatory.

Finally, we also explored how the preference for one of the two tasks in free-choice trials relates to task performance in forced-choice trials, where both tasks had to be performed equally often. Across all four experiments, participants were generally faster in forced-choice trials when responding to the preferred task compared to the non-preferred task (Exp. 1: 664 vs 793 ms, *p* < 0.001; Exp. 2: 664 vs 783 ms, *p* < 0.001; Exp. 3: 713 vs 771 ms, *p* < 0.001; Exp. 4: 620 vs. 646 ms, *p* = 0.004).

## General discussion

In the present experiments, we examined whether voluntary choosing between two tasks (as measured by switching behavior) depends on the previously obtained outcomes (Exp. 1, Exp. 3, Exp. 4: reward vs no reward; Exp. 2: loss vs no loss) and the previously applied processing mode (free versus forced). Generally, we hypothesized that the most recently applied task goal is usually the most highly valued, leading individuals to prefer task repetitions over switches. However, the reception of positive versus negative outcomes may additionally bias how people value task goals, influencing subsequent task choices. Indeed, positively reinforcing task goals after correct performance by random rewards or no-losses generally resulted in fewer subsequent switches. Furthermore, in all experiments, participants generally showed a stronger attachment to a freely-chosen task compared to a forced-chosen task, as reflected in more switches when the previous task was forced rather than free. Interestingly, additional evidence supporting the idea that people differentially value free and forced-choice task goals comes from the finding that participants' switching behavior was more strongly influenced by reward (versus no-reward following freely-chosen tasks compared to forced-chosen tasks) in Exp. 1 and Exp. 3. While this finding suggests that people place a stronger emphasis on outcomes associated with freely chosen task goals, this inflationary effect on free choice behavior seems to be limited to rewards and situations where task choice and task performance more strongly overlap. Specifically, there was no evidence that participants' switching behavior was differentially influenced by losses (versus no loss) following both free and forced task choices in Exp. 2, and also not by reward (versus no reward) when task choice and task performance were separated via a double registration procedure in Exp. 4.

While the effects of rewards on task choice behavior have also been observed in previous voluntary task-switching studies (e.g., Braem, 2017; Fröber & Dreisbach, 2016), to our knowledge, the present study provides the first direct evidence that the immediate impact of recent outcomes can bias subsequent task choice while considering both gain and loss outcomes. Thus, the finding that people tend to switch tasks more often following a loss (versus no loss) and repeat tasks more often following a reward extends previous studies that have shown a similar effect when choosing between different options in gambling-like situations to situations involving the choice between different cognitive tasks (e.g., Elston et al., 2021; Worthy et al., 2013). Assuming that people tend to choose the task with the highest value (e.g., Braun et al., 2018; Spitzer et al., 2024), the present findings suggest that outcome-monitoring processes quickly update the value of different task goals to guide subsequent behavior. As distinct stimuli and responses were used for the two tasks, one could speculate that the reinforcing effect may have also influenced lower-level task-specific processes, such as task-specific response effectors. However, given that outcomes also influence task choices when task performance was separated from task choice in Exp. 4, it seems that outcome-related changes in biasing free task choice behavior depend at least partially on changes in the value of higher-level task representations rather than only on changes in the value of task-specific perceptual input (stimuli) and output (responses).

Moreover, the present study also demonstrates that participants are less likely to switch away from a task when they have the opportunity to choose it compared to when they are forced to choose (cf. Mittelstädt et al., 2024; Qiao et al., 2022). Going beyond previous studies, this specific choice bias remains evident when separating task choice from task performance. Thus, it seems that the act of choosing a task leads to a stronger valuation of that task, increasing the likelihood that its representation will guide subsequent behavior. This finding aligns with research from various domains, consistently demonstrating that options that are freely chosen are more highly valued than those that are not (e.g., Cockburn et al., 2014; Leotti & Delgado, 2014; Patall et al., 2008; Stolz et al., 2020). Moreover, the finding that free-task choices result in more stable behavior, as indicated by fewer voluntary switches, extends previous findings showing that task choice enhances stability by protecting task processing from distracting environmental influences (e.g., Falk et al., 2022; Gendolla et al., 2021).

As outlined in the additional exploratory task choice analyses section, we also investigated whether an overall preference for one of the two tasks could explain why participants tend to repeat a task more often after free-choice compared to forced-choice trials (e.g., in consecutive free-choice trials, participants may consistently select the same task due to an inherent preference for it). The results suggest that stable task preferences alone cannot fully explain why participants show a stronger attachment to a freely-chosen task compared to a forced-chosen task, but these preferences certainly contribute to this effect. Therefore, it appears worthwhile to more directly investigate the role of individual task preferences in voluntary task choice behavior. For example, it is plausible that task preferences vary across trials, suggesting that dynamic changes in task preferences may also partly contribute to the free-task repetition bias. Furthermore, it also seems interesting to consider what drives participants to prefer one task over the other in the first place. Considering that the exploratory analyses also revealed that participants were generally faster when performing their overall preferred task in forced-choice trials, it seems possible that performance optimization might play a critical role (cf. Mittelstädt et al., 2019).

Another novel finding of the present study is that Exp. 1 and Exp. 3 revealed a larger reward-related choice bias after free tasks compared to forced tasks, suggesting that people value more strongly rewarding outcomes of tasks they have freely chosen, as opposed to those that were forced upon them. This finding appears in line with previous studies that have demonstrated increased neural activity, as measured by the feedback-related negativity (FRN, Gehring & Willoughby, 2002), in association with free choice behavior (Mühlberger et al., 2017; Yeung et al., 2005; Zhou et al., 2010). However, in these previous studies, gambling-like choices between options rather than between choice tasks have been investigated and the impact on subsequent behavior has often been neglected (but see Gehring & Willoughby, 2002, for more risky choices after losses). Thus, it remains to be investigated whether similar heightened outcome monitoring on a neuronal level is present when evaluating the outcomes of free versus forced *task* choices, and to what extent this outcome-related neural activity can be linked to subsequent behavior.

Moreover, the specific causes of this free choice reward bias remain unclear and there are several, not mutually exclusive possibilities. First, one might speculate that people perceive higher efficacy in choosing a rewarding task. Specifically, as participants might be more motivated to engage in a task they have freely chosen rather than one imposed on them (e.g., Falk et al., 2022; Gendolla et al., 2021), they may pay more attention to free choice trials and weigh the associated outcomes more heavily. Second, the requirement to choose freely puts people in a state of uncertainty, making them more keen to determine whether they made the “right” choice. As confidence is generally known to affect choice history biases (e.g., Braun et al., 2018; Sanders et al., 2016), receiving a reward for a freely chosen task might particularly increase confidence in that specific task. Third, people may generally value rewards more strongly when obtained from more effortful tasks (cf. Inzlicht et al., 2018; Zentall, 2010), and positive outcomes may be more rewarding when people choose for themselves because choosing a task itself can be a demanding cognitive process (e.g., Kiesel & Dignath, 2017; Schwartz, 2000; Vohs et al., 2018). Fourth, stronger memory representations might be formed for outcomes of free compared to forced choices (e.g., Chambon et al., 2020; Katzman & Hartley, 2020; Murty et al., 2015). Assuming that people guide their task choices based on memory representations, this could also explain why participants rely more heavily on rewarding previous free choice trials when making subsequent free task choices.

When investigating the causes of this reward-specific free-choice bias in future studies, it also seems useful to consider the boundary conditions of this effect. For example, while research on decision-making suggests that people generally weigh avoiding losses more strongly than obtaining gains (loss aversion, see e.g., Chen et al., 2020; Tversky & Kahneman, 1991; Yechiam & Hochman, 2013), it is not clear how such loss versus gain outcome specificity can explain the similar influences of loss versus no-loss on choice behavior in Exp. 2 as a function of free versus forced choice processing.

Furthermore, we can only speculate why, even with rewarding outcomes, Exp. 4 did not reveal evidence for a reward-specific free-choice bias when task choice and task performance screens were separated. For example, one might argue that when separating choice and task processing, participants were better at evaluating that the reward manipulation was not contingent on their behavior, as correct performance was randomly rewarded. The questionnaire data in this experiment align with the idea that the majority of participants realized they had no control over the outcomes.^5^ Furthermore, it is interesting to note that exploratory analyses revealed that participants with lower forced switch costs in Experiment 4 did not exhibit higher switch rates, unlike in the other three experiments.^6^ This suggests that task choices in this experiment were not related to actual performance. One might speculate that reward-specific free-choice bias only emerges in environments where task choices are at least partially linked to performance, as performance efficacy—that is, the potential for increased performance to lead to better outcomes—might play a critical role in perceived control over outcomes. Thus, in future studies, it may be useful to either induce some perceived control (e.g., via a cover story), some actual choice-reward contingencies or (partial) performance-based rewards to better understand how free- and forced-task goal outcomes are evaluated.

Finally, as illustrated by the additional raincloud plots in Appendix B, there was generally quite a bit of individual variability in task choice behavior in the specific conditions. Thus, it also seems useful to consider interindividual differences in task choice behavior as a function of outcome (and processing mode) in future research. For example, Mittelstädt et al. (2021) showed in a purely voluntary task switching environment that some people rather preplan their task choices across several trials, while others decide spontaneously in each trial which task to choose. One might suspect that individuals following spontaneous task choice strategies are more sensitive to environmental cues, such as the present outcome manipulation.

In sum, this study began with the premise that people choose tasks based on their perceived value and it offers new insights into how task values are updated by outcomes and processing modes, thereby influencing subsequent voluntary task-switching behavior. Specifically, the findings indicate that people tend to repeat tasks more frequently following positive outcomes rather than negative ones and they show a preference for tasks they have chosen themselves over those imposed upon them. This suggests that both positive outcomes and free choice enhance the perceived value of task goals. Furthermore, individuals may sometimes overestimate the value of outcomes linked to tasks they have freely chosen compared to those imposed upon them. This was evident in Experiments 1 and 3, where outcomes had a stronger impact on task-switching behavior following freely chosen tasks than forced tasks. Given that we can currently only interpret the findings against a rather vague theoretical assumption that task values drive task choice behavior, it will be crucial for future studies to investigate how outcome evaluation for freely chosen and forced task goals can be conceptualized using more detailed theoretical, ideally computational, models. Considering that tasks performed in many real-world situations often differ in their degree of freedom, understanding how choices along a continuum from self-determined to externally-determined influence our behavior might also be useful for applied contexts.

## Figures and Tables

**Fig. 4:**
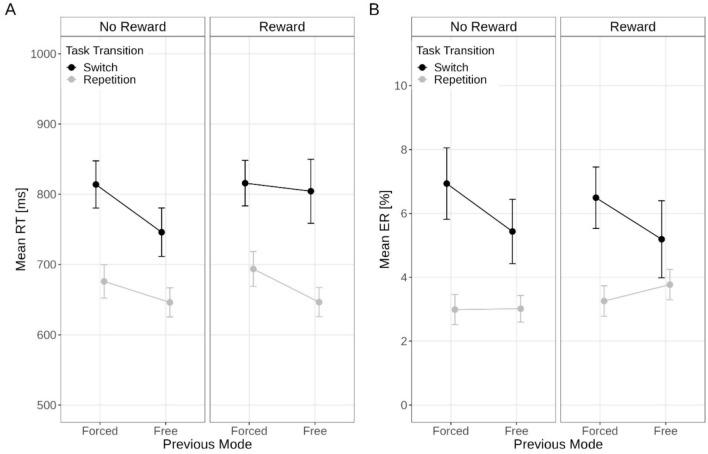
Mean reaction times (RTs) and mean percentage error (PE) in Experiment 1 as a function of task transition (switch, repetition), previous mode (previous-free, previous-forced) and feedback (reward, no-reward) for current free-choice tasks

**Fig. 5:**
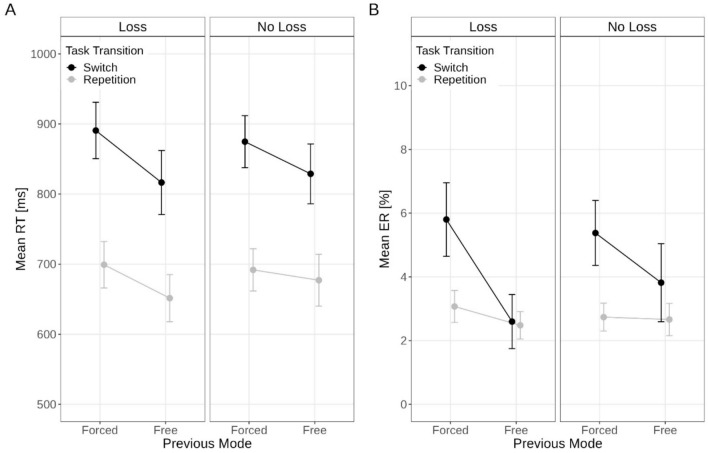
Mean reaction times (RTs) and mean percentage error (PE) in Experiment 2 as a function of task transition (switch, repetition), previous mode (previous-free, previous-forced) and feedback (reward, no-reward) for current free-choice tasks

**Fig. 6:**
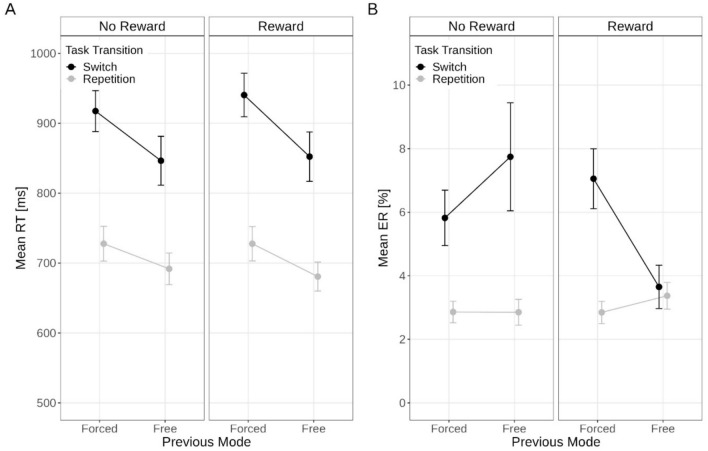
Mean reaction times (RTs) and mean percentage error (PE) in Experiment 3 as a function of task transition (switch, repetition), previous mode (previous-free, previous-forced) and feedback (reward, no-reward) for current free-choice tasks

**Fig. 7:**
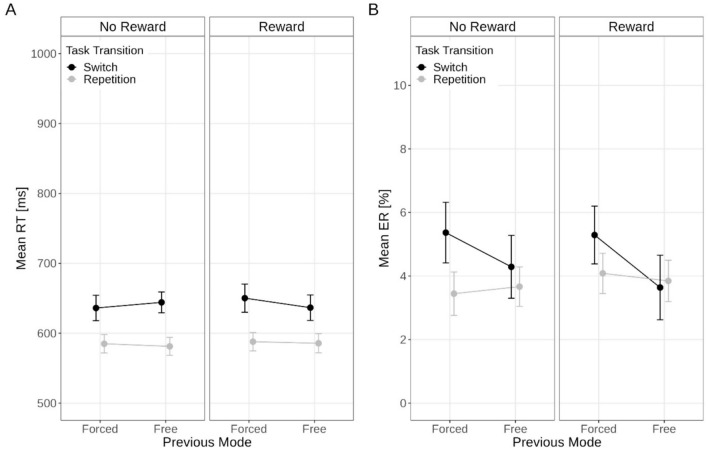
Mean reaction times (performance-RTs) and mean percentage error (performance-PE) for task performance and mean reaction time (choice-RTs) for task choice in Experiment 4 as a function of task transition (switch, repetition), previous mode (previous-free, previous-forced) and feedback (reward, no-reward) for current free-choice tasks

**Fig. 8:**
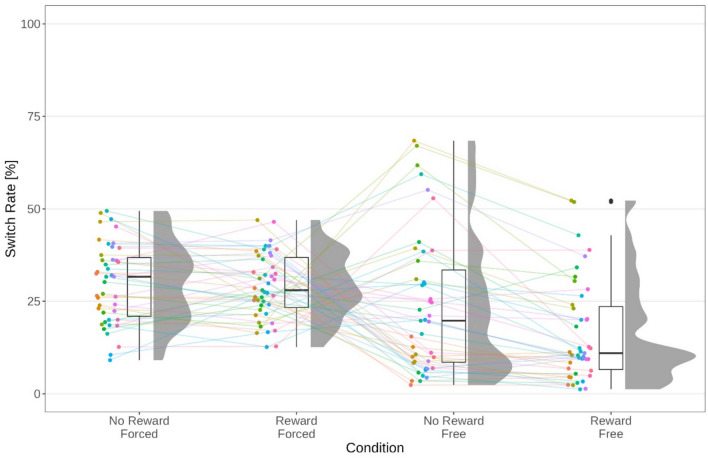
Raincloud plots showing percentage of task switches as a function of previous mode (free, forced) and feedback (reward, no-reward) for Experiment 1

**Fig. 9:**
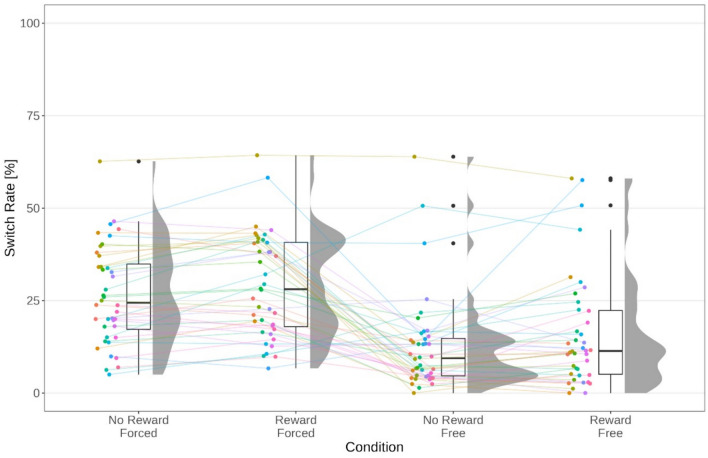
Raincloud plots showing percentage of task switches as a function of previous mode (free, forced) and feedback (loss, no-loss) for Experiment 2

**Fig. 10:**
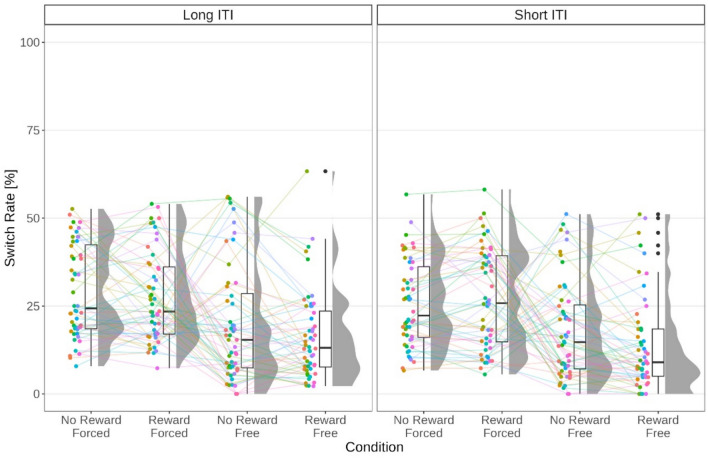
Raincloud plots showing percentage of task switches as a function of previous mode (free, forced), feedback (reward, no-reward) and intertrial interval (ITI; short, long) for Experiment 3

**Fig. 11:**
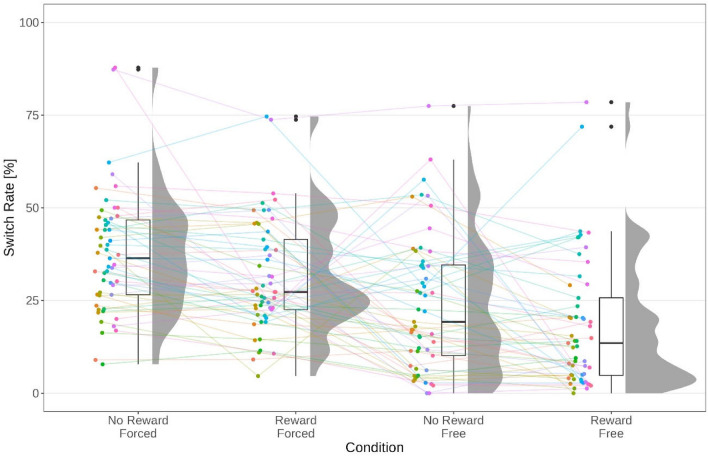
Raincloud plots showing percentage of task switches as a function of previous mode (free, forced) and feedback (reward, no-reward) for Experiment 4

## Data Availability

Preregistrations, raw data and analysis scripts for the task choice results reported in the main text (including variable descriptions) are available via the Open Science Framework at https://osf.io/vmh3d/?view_only=1cd4785a16544d568a2e54d4dccbc642.

## Appendix A

To gain a better understanding of the influences of our experimental factors on free choice behavior, we also investigated task performance in terms of reaction times (RT) and percentage errors (PE) for free-choice trials in all experiments as well as task choice-RTs in free-choice trials in Experiment 4. Thus, in this Appendix, we present additional analyses of free-choice task performance (RT and PE). We applied the same data preparation procedure as described in the main text, but this time we included error trials for the analysis of PE.

### Experiment 1

We conducted a 3-way within-subject ANOVAs on the mean RTs and mean PEs of free-choice trials (see Fig. 4), considering the factors of previous mode (previous-free versus previous-forced), previous feedback (reward versus no-reward), and task transition (switch versus repetition).

#### Mean RT

There was a significant main effect of task transition, indicating switch costs of 130 ms, *F*(1, 42) = 75.30, *p* < 0.001, η_p_^2^ = 0.64. The main effect of previous mode was also significant, reflecting costs of switching between processing modes as participants were 39 ms faster after free- compared to forced-choice responses, *F*(1, 42) = 17.56, *p* < 0.001, η_p_^2^ = 0.29. The main effect of previous reward was also significant, reflecting 20 ms faster free-choice processing after no-reward compared to reward *F*(1, 42) = 17.56, *p* < 0.001, η_p_^2^ = 0.29. Finally, there was a significant three-way interaction, *F*(1, 42) = 4.27, *p* = 0.045, η_p_^2^ = 0.09.

Separate 2-way ANOVAs were conducted for previous mode free and forced trials. When the previous mode was forced, there were was only a significant main effect of task transition (i.e., switch costs), *F*(1, 42) = 115.03, *p* < 0.001, η_p_^2^ = 0.73 (all other *p* > 0.310, all η_p_^2^ < 0.03). When the previous mode was free, there was a like main effect of task transition, *F*(1, 42) = 34.13, *p* < 0.001, η_p_^2^ = 0.34, but the main effect of previous reward was also significant, reflecting faster responses when no reward compared to reward was obtained, *F*(1, 42) = 4.90, *p* = 0.032, η_p_^2^ = 0.10. Moreover, there was a significant interaction, indicating that task switch costs were larger after having received reward than no reward (172 ms and 144 ms), *F*(1, 42) = 4.46, *p* = 0.041, η_p_^2^ = 0.10.

#### Mean PE

There was only a significant main effect of transition, indicating switch costs of 2.8%, *F*(1, 42) = 25.51, *p* < 0.001, η_p_^2^ = 0.38 (all other *p* > 0.143, all other η_p_^2^ < 0.06).

### Experiment 2

For the corresponding ANOVAs in Experiment 2 (see Fig. 5), three additional participants had to be excluded due to a lack of trials in at least one cell. Note that excluding these participants for the task choice analyses reported in the main text does not affect the results.

#### Mean RT

There was a significant main effect of transition, indicating switch costs of 173 ms, *F*(1, 36) = 90.28, *p* < 0.001, η_p_^2^ = 0.71. There was also a significant main effect of previous mode, indicating 46 ms slower responses after forced- compared to freely-chosen tasks, *F*(1, 36) = 13.99, *p* = 0.001, η_p_^2^ = 0.28. Except for a marginal significant two-way interaction between previous reward and previous mode, *F*(1, 36) = 3.03, *p* = 0.090, η_p_^2^ = 0.08, no other effects were significant (all other *p* > 0.285, all other η_p_^2^ < 0.04). As can be seen in Fig. 5, when the previous mode was free, responses were 19 ms faster after no-loss compared to loss, whereas when the previous mode was forced, responses were 13 ms slower after loss compared to no-loss.

#### Mean PE

There was a significant main effect of transition, indicating switch costs of 1.7%, *F*(1, 36) = 8.01, *p* = 0.008, η_p_^2^ = 0.18. Moreover, there was a significant main effect of previous mode, indicating 1.3% more errors ms after forced- compared to freely-chosen tasks, *F*(1, 36) = 11.39, *p* = 0.002, η_p_^2^ = 0.24. Finally, there was a significant interaction between task transition and previous mode, *F*(1, 36) = 5.20, *p* = 0.029, η_p_^2^ = 0.13 (all other *p* > 0.328, all other η_p_^2^ < 0.04). As can be seen in Fig. 5, task switch costs were larger when the previous mode was forced compared to free.

### Experiment 3

We collapsed across the two ITI conditions^7^ and conducted again 3-way within-subject ANOVAs on the mean RTs and mean PEs of free-choice trials (see Fig. 6), considering the factors of previous mode (previous-free versus previous-forced), previous feedback (reward versus no-reward) and task transition (switch versus repetition). Note that one participant had to be excluded due to a lack of trials in one cell condition.

#### Mean RT

There was a significant main effect of task transition, indicating switch costs of 182 ms, *F*(1, 50) = 134.70, *p* < 0.001, η_p_^2^ = 0.73. The main effect of previous mode was also significant, reflecting costs of 60 ms after forced- compared to free-choice responses, *F*(1, 50) = 37.77, *p* < 0.001, η_p_^2^ = 0.29. Finally, there was a marginal significant two-way interaction between previous mode and task transition, *F*(1, 50) = 3.34, *p* = 0.074, η_p_^2^ = 0.06 (all other *p*s > 0.360, all other η_p_^2^ < 0.03). Task switch costs were slightly larger when the previous mode was forced than free.

#### Mean PE

There was a significant main effect of transition, indicating switch costs of 3.0%, *F*(1, 50) = 38.54, *p* < 0.001, η_p_^2^ = 0.44. Moreover, there was a significant interaction between previous reward and previous mode, *F*(1, 50) = 4.45, *p* = 0.040, η_p_^2^ = 0.08, which was further modulated by task transition, *F*(1, 50) = 8.48, *p* = 0.005, η_p_^2^ = 0.14.

Separate 2-way ANOVAs were conducted for previous mode free and forced trials. When the previous mode was forced, there were was only a significant main effect of task transition (i.e., switch costs), *F*(1, 50) = 28.96, *p* < 0.001, η_p_^2^ = 0.37 (all other *p* > 0.259, all η_p_^2^ < 0.04). When the previous mode was free, there was also a main effect of task transition, *F*(1, 50) = 9.04, *p* = 0.004, η_p_^2^ = 0.15, a marginal significant main effect of reward, *F*(1, 50) = 3.29, *p* = 0.076, η_p_^2^ = 0.06, and a significant interaction, *F*(1, 50) = 6.12, *p* = 0.017, η_p_^2^ = 0.11. Task switch costs were smaller after having received reward than no reward (0.3% and 4.9%).

### Experiment 4

We first conducted 3-way within-subject ANOVAs on the mean RTs and mean PEs of free-choice trials related to the task performance screen (see Fig. 7), considering the factors of previous mode (previous-free versus previous-forced), previous feedback (reward versus no-reward) and task transition (switch versus repetition). Moreover, we conducted a similar ANOVA on the mean RTs of free-choice trials related to the task choice screens (see Fig. 7). Note that three additional participants had to be excluded due to a lack of trials in one cell condition.

#### Mean performance-RT

There was only a significant main effect of task transition, indicating switch costs of 57 ms, *F*(1, 49) = 54.77, *p* < 0.001, η_p_^2^ = 0.53 (all other *p*s > 0.149, all η_p_^2^ < 0.045).

#### Mean performance-PE

There were no significant effects (all *p* > 0.145, all η_p_^2^ < 0.05).

#### Mean choice-RT

There was a significant main effect of task transition, indicating 51 ms slower responses when choosing to switch compared to repeat tasks (509 ms versus 458 ms), *F*(1, 49) = 47.53, *p* < 0.001, η_p_^2^ = 0.49. Moreover, there was a significant main effect of previous mode, indicating 48 slower responses when the previous mode was forced compared to free, *F*(1, 49) = 64.44, *p* < 0.001, η_p_^2^ = 0.57. Finally, there was a significant main effect of previous feedback, indicating 14 ms slower responses when reward compared to when no-reward was obtained in the previous trial, *F*(1, 49) = 7.94, *p* = 0.007, η_p_^2^ = 0.14(all other *p*s > 0.149, all η_p_^2^ < 0.04).

### Summary of Appendix A

First, in all experiments substantial task switch costs were observed, indicating that participants represented distinct tasks, and switching between them required cognitive effort and time. Secondly, there were additional costs when switching (previous mode: forced) rather than repeating (previous mode: free) the processing mode in Experiments 1–3. These differences suggest that free- and forced-task choice goals are differentially internally represented, so switching between such goals takes additional time (even when repeating the same task). Interestingly, for Experiment 4, where task choice and task performance were separately measured, these processing mode switch costs were present in choice RTs; that is, it took participants longer to choose between the two tasks when they were forced to choose a task in the previous trial. Thus, this finding provides new evidence that choosing itself (without any task processing) can be more time-consuming and/or demanding when participants recently could not choose. Third, while there was some evidence in Experiments 1–3 that the previous outcomes influenced subsequent task performance, these influences were not systematic. For example, when the previous mode was free, in Exp. 1 task switch costs in RTs were larger after a reward than no reward, whereas in Exp. 3 task switch costs in error rates were smaller after a reward than no reward. Thus, based on this evidence, it is not clear to what extent participants adapted their task performance to the outcome manipulation. Moreover, as in Exp. 4, choice RTs were smaller when reward compared to no reward was obtained, it may be that the influence in the other three experiments primarily reflects changes in the duration of choosing rather than actually performing the task.

## Appendix B

Figures 8, 9, 10, and 11 show raincloud plots (see Allen et al., 2019) for each experiment, illustrating individual percentages of switches in the experiment-specific conditions. Note that the R-package ggplot2 (Wickham, 2011), combined with ggdist (Kay, 2023), was used to create these plots.

## Footnotes

1 Note also that in the studies by Mittelstädt et al. (2024) and Qiao et al. (2022), there has been no investigation into whether this specific free-task choice repetition bias remains when dissociating the processes underlying task choice from task execution. This issue will be addressed in Experiment 4 of the present study.

2 The sample size in all experiments was somewhat arbitrarily set based on sample sizes used in previous studies (e.g., Jurczyk et al., 2019; Mittelstädt et al., 2022) while taking into account practical constraints (e.g., participants availabilities) and empirical constraints (e.g., power). For example, a power analysis using the R-package Superpower (Lakens & Caldwell, 2021) indicated that with a sample size of 50 participants we would have over 80% power with a significance level of 5% to detect an effect of at least η_p_^2^ = 0.14 for the 2 × 2 interaction of feedback (reward, no-reward) and processing mode (free, forced). The effect sizes of previous feedback (η_p_^2^ = 0.27) and its interaction with the previous mode (η_p_^2^ = 0.23) in Experiment 1 were rather large. A power analysis indicated that we would have over 80% power with a significance level of 5% to detect an effect of at least η_p_^2^ = 0.22 with 31 participants. However, to account for potential dropouts and the possibility that the effect may be smaller due to the loss manipulation, we decided to test another 50 participants in Experiment 2.

3 We also explored potential differences in choosing between the two tasks, as well as task-specific performance differences. Across all experiments, there was generally a tendency to select the color task more often than the letter task (Exp. 1: 51.4% of color task responses, Exp. 2: 62.3%, Exp. 3: 59.3%, Exp. 4: 56.4%). Moreover, participants were generally also faster in responding to the color task than to the letter task (Exp. 1: 22 ms speed advantage, Exp. 2: 76 ms, Exp. 3: 34 ms, Exp. 4: 38 ms), without any meaningful or consistent differences in error rates.

4 Note that we also conducted a mixed ANOVA with the within-subject factor feedback (yes versus no, depending on whether participants received gains or losses) and previous mode (free versus forced), and the between-subject factor experiment (Exp. 1, Exp. 2). This ANOVA revealed a significant three-way interaction, *F*(1, 89) = 12.70, *p* =0 .001, η_p_^2^ = 0.12, suggesting that the type of feedback (gains or losses) indeed differentially interacted with previous mode.

5 For all experiments, we also investigated whether the present choice pattern remained stable over the experiment by reanalyzing the data separately for the first and second halves of each experiment. In general, the qualitative pattern was rather similar, but there were some hints that may suggest that participants are more strongly affected by the outcome manipulation in the first half than in the second half. Specifically, for Exp. 2, the main effect of outcome was only significant in the first half (*p* =0 .001) but not the second half (*p* = 0.112). Moreover, in Exp. 3, the interaction between previous outcome and previous mode was only significant in the first half (*p* =0 .026) but not the second half (*p* =0 .191). While the interaction was significant for both the first (*p* <0 .001) and second half in Exp. 1 (*p* = 0.041), the differences were more prominent in the first half.

6 Specifically, the correlations of mean forced switch costs and individual switch rates (averaged across conditions) were: *r*(43) = 0.56 (*p* <0 .001) in Exp. 1, *r*(39) = 0.34 (*p* = 0.029) in Exp. 2, *r*(52) = 0.50 (*p* < 0.001) in Exp. 3, and *r*(53) = 0.04 (*p* =0.759) in Exp. 4.
